# Operational research in malawi: making a difference with cotrimoxazole preventive therapy in patients with tuberculosis and HIV

**DOI:** 10.1186/1471-2458-11-593

**Published:** 2011-07-27

**Authors:** Anthony D Harries, Rony Zachariah, Rhehab Chimzizi, Felix Salaniponi, Francis Gausi, Henry Kanyerere, Erik J Schouten, Andreas Jahn, Simon D Makombe, Frank M Chimbwandira, James Mpunga

**Affiliations:** 1 International Union against Tuberculosis and Lung Disease, Paris, France; 2 London School of Hygiene and Tropical Medicine, London, UK; 3 Medecins sans Frontieres, Medical Department, Operational Research Unit, Brussels Operational Center, Luxembourg, Luxembourg; 4 Management Sciences for Health, Accra, Ghana; 5 KNCV Kenya Office, PO Box 27789, Nairobi, Kenya; 6 National Tuberculosis Control Programme, Community Health Science Unit, Lilongwe, Malawi; 7 Management Sciences for Health, Lilongwe, Malawi; 8 Department of HIV and AIDS, Ministry of Health, Malawi; 9 ITECH, Malawi and University of Washington, Seattle, USA

**Keywords:** Operational research, cotrimoxazole preventive therapy, tuberculosis, HIV/AIDS, Malawi, Africa

## Abstract

**Background:**

In Malawi, high case fatality rates in patients with tuberculosis, who were also co-infected with HIV, and high early death rates in people living with HIV during the initiation of antiretroviral treatment (ART) adversely impacted on treatment outcomes for the national tuberculosis and ART programmes respectively. This article i) discusses the operational research that was conducted in the country on cotrimoxazole preventive therapy, ii) outlines the steps that were taken to translate these findings into national policy and practice, iii) shows how the implementation of cotrimoxazole preventive therapy for both TB patients and HIV-infected patients starting ART was associated with reduced death rates, and iv) highlights lessons that can be learnt for other settings and interventions.

**Discussion:**

District and facility-based operational research was undertaken between 1999 and 2005 to assess the effectiveness of cotrimoxazole preventive therapy in reducing death rates in TB patients and subsequently in patients starting ART under routine programme conditions. Studies demonstrated significant reductions in case fatality in HIV-infected TB patients receiving cotrimoxazole and in HIV-infected patients about to start ART. Following the completion of research, the findings were rapidly disseminated nationally at stakeholder meetings convened by the Ministry of Health and internationally through conferences and peer-reviewed scientific publications. The Ministry of Health made policy changes based on the available evidence, following which there was countrywide distribution of the updated policy and guidelines. Policy was rapidly moved to practice with the development of monitoring tools, drug procurement and training packages. National programme performance improved which showed a significant decrease in case fatality rates in TB patients as well as a reduction in early death in people with HIV starting ART.

**Summary:**

Key lessons for moving this research endeavour through to policy and practice were the importance of placing operational research within the programme, defining relevant questions, obtaining "buy-in" from national programme staff at the beginning of projects and having key actors or "policy entrepreneurs" to push forward the policy-making process. Ultimately, any change in policy and practice has to benefit patients, and the ultimate judge of success is whether treatment outcomes improve or not.

## Background

Operational research may be defined as the search for knowledge on strategies, tools or interventions which leads to improved programme performance and/or health service delivery [1]. In 1996, the Malawi National Tuberculosis Control (NTP) Programme embraced the concept of operational research and started a research programme that translated directly into several improvements in policy and practice [2,3]. Before this year, TB control activities had not been going well, quarterly supervision had declined and funding was an issue. The Department for International Development, UK, (DFID) pledged support for TB programme activities, such as procurement of drug and consumable supplies and routine quarterly supervision, and for operational research with the latter activity being used within the programme to collect data on weaknesses and to implement interventions to solve the challenges that it faced. This support by a new donor at that time was welcomed by the Malawi Ministry of Health.

Between 1996 and 2004, other donors such as the Norwegian Agency for International Cooperation (NORAD) and the Royal Netherlands Tuberculosis Association (KNCV) came on board to support activities within the Malawi NTP that included operational research as an integral part of programme activities. For the research, a partnership was set up whereby research ideas from within the National TB Programme, from local institutions (such as the Malawi Medical School, non-governmental organizations such as Medecins sans Frontieres and the National AIDS Programme) and from international organizations (such as the World Health Organization, The International Union Against Tuberculosis and Lung Disease and the Liverpool School of Tropical Medicine) were discussed and endorsed at the six-weekly meetings of the Malawi TB Programme Management Group. After priorities were established, research activities were then implemented by the various stakeholders, although many were planned, initiated, completed and published within the Malawi TB Programme itself.

The guiding principles that under-pinned the research agenda included i) defining the programme objectives, ii) identifying constraints that prevented objectives being met, and iii) asking research questions around those constraints to try and find solutions that would allow programme objectives to be achieved. In 2004, when scale up of antiretroviral therapy (ART) started in Malawi, operational research based around the same guiding principles was also used to inform policy and practice around the delivery of ART.

As an example of how this can work at the national level for TB and ART programmes and how guiding principles of operational research are put into practice, we describe the operational research that was carried out in Malawi with cotrimoxazole preventive therapy (CPT), initially in HIV-infected tuberculosis (TB) patients and then all HIV-infected patients starting ART. We outline the steps that were taken to translate these findings into policy and practice, and for both TB patients and HIV-infected patients starting ART show how the implementation of CPT made a difference and reduced death rates. We finally draw on general lessons that can be learnt for other settings and interventions, and suggest that such outcome indictors of deaths prevented or lives saved are the true measure of whether operational research in programme settings is useful or not.

## Discussion

### Effect of HIV on increasing death rates and reducing cure rates in the Malawi TB Control Programme

Malawi is a small country in southern Africa with a current population of about 13 million. In the 1980s, the country had one of the first "model" TB control programmes, a harbinger of the "DOTS" strategy, with about 4,000 registered cases per annum and cure rates in new smear-positive pulmonary TB patients at or higher than 90% [2,3]. These excellent treatment success rates were not to last. In December 1985, the first AIDS case was reported in the country, and within ten years HIV-prevalence in the adult population had soared to 14% [4]. Despite a well functioning NTP, annual case notifications spiralled out of control to reach 25,000 by the mid-1990s, which were associated with HIV co-infection rates of 75% [2,3].

Accompanying the increase in case notifications was a rapid increase in case fatality, which was reported from the programme setting and as well as from carefully monitored cohorts of patients, the case fatality also being strongly associated with HIV [2,3,5,6]. This had a major impact on cure rates in new smear-positive PTB patients which plummeted to their nadir in 1996 at 63% [2,3]. It became apparent in the mid-1990s that "DOTS" on its own was insufficient to control the TB epidemic, and HIV-associated interventions would be required if death rates were to be reduced.

### Need for operational research to assess interventions to reduce death rates in TB patients

Two randomised controlled trials in Cote d'Ivoire assessing the effect of cotrimoxazole in HIV-infected adults were published in 1999. The first showed a decrease in morbidity in HIV-infected adults [7], while the second conducted in HIV-infected patients with TB showed a significant reduction in mortality [8]. These studies persuaded the Joint United Nations Programme on AIDS (UNAIDS) to issue provisional recommendations in 2000 that all people living with HIV (PLHIV) in Africa who were symptomatic should receive CPT as part of a standard package of care [9].

The Cote d'Ivoire trial and the UNAIDS recommendations had important implications. At the time, there were three randomised controlled trials on CPT taking place in Malawi, Senegal and Cape Town, and all of these were prematurely stopped due to ethical considerations that evidence of efficacy was now established. However, the Malawi Ministry of Health (MoH) was reluctant to embark on a national policy of CPT for all PLHIV because of concerns about differences in commonly occurring disease pathogens and cotrimoxazole resistance rates between West and Central Africa. Furthermore, there were fears that widespread use of CPT would encourage cross-resistance to sulphadoxine-pyrimethamine which, at the time, was the national first line anti-malarial treatment for *Plasmodium falciparum *[10]. The Malawi MoH therefore encouraged and endorsed district operational research to gather national evidence to support or refute the use of CPT.

### Operational research on offering HIV testing and cotrimoxazole to TB patients in Malawi and the initial policy decision

Two district-based operational research studies were undertaken and completed in Thyolo, the southern region, and Karonga, the northern region of Malawi [11,12]. The aim of the two studies was similar, namely to evaluate the feasibility and effectiveness of a package of HIV testing and CPT offered to TB patients registered under routine programme conditions. Mortality during anti-TB treatment was documented in all TB patients offered this package and registered during a 12-month period, and compared with mortality in all TB patients not offered the package and registered during a 12-month period the year before - namely, "historical controls". Active household tracing of patients was undertaken in both districts to ensure that mortality data were reliable.

A total of 2,703 TB patients were studied in both groups and in the two districts. In Thyolo, overall case fatality significantly declined from 36% in the control group to 28% in the intervention group, and in Karonga overall case fatality was also significantly reduced from 37% to 29%. The number of TB patients needing HIV testing and CPT to prevent one death during the course of anti-TB treatment was calculated at 12.5 in each district. In Blantyre district, a further study was conducted in 579 HIV-infected TB patients comparing two different doses of CPT and comparing case fatality rates with those observed in the National TB cohort and a previous TB cohort in whom a large majority had been tested for HIV and carefully followed to the end of TB treatment [13]. Case fatality was significantly reduced in patients offered CPT, and there was no difference in outcomes between patients offered CPT 480 mg daily and those offered 960 mg daily.

The results of these district operational research studies were presented at a large stakeholders' meeting convened by the Malawi MoH in October 2002. This meeting was organised by certain key actors within the TB Programme - so called "policy entrepreneurs"(see Table 1) - who ensured that the policy-making process remained on the agenda and moved forward. Important policy decisions were made at the end of that meeting [14]. The package of HIV testing and CPT was to be continued in the three districts in TB patients, and the intervention was to be scaled up to all TB patients country wide in a phased approach over three years. This was to be accompanied by appropriate guidelines, a training package and responsibility for procurement and distribution of CPT staying in the hands of the Malawi NTP. The uptake of the intervention was to be carefully monitored along with treatment outcomes, and further operational research was to be conducted as necessary to answer relevant questions arising from the field. As there was no evidence to support the benefit of HIV testing and CPT in PLHIV who did not have TB, the intervention was to be used only for HIV-infected TB patients until such time as additional evidence of benefit in PLHIV without TB was available.

**Table 1:** "Policy entrepreneurs" in the context of the Malawi National TB Programme

These are senior people within the National TB Programme (TB Programme Director and National TB Advisor responsible for operational research), who are well connected with senior personnel in the Ministry of Health and other actors in the health sector (for example, the Medical School)
They are responsible for the overall TB operational research programme and provide direction to the research questions and research implementation in the field
They assess the outcomes of the research and decide how this may influence policy within the context of the TB Programme and the wider health sector: this is discussed within programme management group meetings
Once decisions are made about the way forward, they assume responsibility for initial discussions with senior people in the Ministry of Health (for example, director of preventive services, secretary for health)
They take responsibility for the forthcoming policy meetings, and act as the secretariat for the organization and chairmanship of the meetings and for writing the minutes
They take responsibility for drafting new policy, and once this is agreed for dissemination country wide

### Scaling up HIV counselling and cotrimoxazole for TB patients, further operational research and impact on TB programme performance

As a result of the policy decision from the MoH, the Malawi NTP together with the National AIDS Commission developed a 3-year plan to expand HIV-TB activities [14]. Soon after this plan was approved in late 2002, a country-wide situational assessment was carried out to assess the state of HIV/AIDS and joint HIV/TB services in hospitals, health centres and clinics throughout the country and to identify facilities to be included in the first phase of HIV testing and CPT implementation. National guidelines were developed that included how the package was to be administered, contraindications to CPT, doses for adults and children, management of adverse effects, logistics of providing CPT and finally how to use the new HIV testing and CPT registers for monitoring and evaluation. These registers were prepared and printed, and were used alongside TB patient registers. A training package was developed and a structured plan put in place to brief and train all TB registration facilities over a three-year period

CPT scale up started in 2003 at 15 facilities. An early review of the first 3 months' activities was carried out and proved invaluable in identifying challenges and solving misunderstandings [14]. Further operational research was also undertaken to answer pertinent questions. A study in Thyolo district showed that adherence to CPT in rural areas was excellent based on verbal verification of drug intake, physical verification of pill count balance and urine trimethoprim detection by gas chromatography and mass spectrometry [15]. Despite good medication adherence, research also demonstrated a growing increase of faecal *Escherichia coli *resistance to cotrimoxazole in HIV-infected TB patients receiving the drug, which prompted some concerns about the long term protective benefits of such chemoprophylaxis [16]. During scale up, the Malawi NTP was responsible for the administration of CPT during anti-TB treatment, but once this was completed patients were referred back to general health services to receive medication. Operational research documented that the majority of patients continued receiving CPT from health centres, although drug stock-outs and transport costs to health centres to collect drugs lead to interruptions of prophylaxis [17].

Routine data from the Malawi NTP showed that between 2002 and 2008 there was a significant increase in HIV testing amongst TB patients with the majority of HIV-positive patients being started on CPT (Table 2a). Treatment outcomes in new smear-positive pulmonary TB patients gradually improved, and by 2008, the global cure rate target of 85% was reached for the first time in 20 years since the start of the HIV/AIDS epidemic (Table 2b).

**Table 2:** National Tuberculosis case finding and treatment outcome data in Malawi between 2002 and 2008

2 (a): Case Notifications, HIV testing and Cotrimoxazole Preventive Therapy (CPT)							
	**2002**	**2003**	**2004**	**2005**	**2006**	**2007**	**2008**

TB case notifications	27,531	28,234	27,000	27,610	27,105	25,966	25,688
Number HIV tested (%)	2130 (8%)	3983 (14%)	6681 (25%)	12243 (44%)	17,253 (64%)	21,551 (83%)	21557 (84%)
Number HIV-positive (%)	1,630 (77%)	2,734 (69%)	4,804 (72%)	8,453 (69%)	12,064 (70%)	15,491 (72%)	13,677 (63%)
Number started CPT (%)	Not known	2,349 (86%)	4,649 (97%)	8,073 (96%)	11,244 (93%)	13,779 (89%)	13,148 (96%)

**2 (b): Treatment outcomes in new smear-positive PTB patients evaluated nationally for outcomes**							

	2002	2003	2004	2005	2006	2007	2008

New smear-positive PTB patients evaluated	7,693	7,603	8,021	7,965	7,955	8065	7632
Treatment success (%)	5,572 (72%)	5,650 (74%)	6,082 (76%)	6,178 (78%)	6,369 (80%)	6707 (83%)	6534 (86%)
Death (%)	1,500 (19%)	1,410 (19%)	1,387 (17%)	1,265 (16%)	1,018 (13%)	739 (9%)	574 (7.5%)
Other outcomes (%)	621 (9%)	543 (7%)	552 (7%)	522 (6%)	568 (7%)	619 (8%)	524 (6.5%)

Legend: other outcomes = default, transfer out, failure. *[the data were obtained from annual NTP reports]*

### Scale up of antiretroviral therapy and the problem of early death rates

In 2004, the country embarked on rapid scale up of antiretroviral therapy (ART), supported financially through the Global Fund to fight AIDS, TB and malaria (GFATM) and implemented through a public health approach based on TB-DOTS principles [18,19]. One of the major problems encountered in the first years of ART scale up was high early mortality- defined as deaths during the first 6 months of treatment. This finding was similar to other countries all over sub-Saharan Africa [19,20]. In the quarterly reports produced by the HIV Department, a consistent finding was that two thirds of all patients known to have died on ART did so in the first three months of treatment. Measures to reduce early mortality were urgently needed.

### Operational research on cotrimoxazole to reduce early death rates in HIV-infected persons starting antiretroviral therapy and policy decision

Anecdotal experience suggested that CPT given before or at the start of ART reduced early death rates, and operational research was carried out to provide more evidence for this intervention. Comparisons of 6-month mortality with data obtained from ART registers and medical records were made between 6 facilities providing ART without CPT and 5 facilities providing ART with CPT [21]. The 6-month mortality rate was significantly lower at ART-CPT sites (10.7%) compared with ART sites alone (18%) [6-month mortality risk reduction = 41%, p = 0.0013], with survival differences apparent as early as 40 days after the start of ART. These data were consistent with subsequent reports from other African countries demonstrating a synergistic effect of CPT with ART, especially in the early months of treatment [22,23]. The Malawi data prompted the HIV Department of the MoH, again through "policy entrepreneurs", to convene a national stakeholders meeting to re-examine the use of CPT in PLHIV.

At the national stakeholders meeting in 2005, new evidence on CPT was reviewed, particularly studies that had been carried out in other sub-Saharan African countries [24-27], which included the joint WHO/UNAIDS/UNICEF statement on use of cotrimoxazole as prophylaxis in HIV-exposed and HIV-infected children [28]. Evidence showed that CPT was associated with a 25%-46% reduction in mortality in PLHIV in sub-Saharan Africa, even in areas with high bacterial resistance to the antibiotic. CPT was also associated with fewer hospitalisations, weight gain, a rise in CD4-lymphocyte counts and a decrease in HIV viral loads. Efficacy was maintained over 1-2 years of follow-up. There were few adverse reactions and high levels of adherence were documented. In summary, CPT appeared to be a safe, cheap and readily available anti-microbial agent, which could extend and improve the quality of life of PLHIV. The earlier concerns about widespread use of CPT increasing resistance of *Plasmodium *falciparum to sulfadoxine-pyrimethamine were partially allayed by studies in children in Mali [29].

There was therefore unanimous agreement to modify the current national recommendations for CPT, and for Malawi to adopt a policy that CPT be provided free of charge to adults and children living with HIV/AIDS as part of a minimum package of care [30] (see Table 3). Malawi's policy and guidelines were in line with those subsequently released by WHO in 2006 [31].

**Table 3:** Policy Guidelines for Cotrimoxazole Preventive Therapy in Malawi (2005)

In Adults:
Cotrimoxazole should be offered to the following HIV-positive adults (aged 15 years and above):
• All persons with symptomatic HIV disease (WHO Clinical Stage 2,3 and 4)
• All persons who have a CD4-lymphocyte count of 500/mm^3 ^or less, regardless of symptoms
• Pregnant women after the first trimester who are symptomatic or have a CD4-lymphocyte count < 500/mm^3 ^
*Note: In adults there is not enough evidence to recommend cotrimoxazole to HIV-positive adults who are asymptomatic (i.e., WHO Clinical Stage 1). However, if evidence is forthcoming in the future to support a change, then this recommendation will be re-examined. It is also felt that the threshold of CD4-count of 500 cells/mm^3 ^may be too high, but it is agreed to stay with this threshold as it is similar to that recommended by the World Health Organization. Again, if evidence is forthcoming in the future that this threshold is too high, the recommendation will be re-examined*
In Children:
Cotrimoxazole should be offered to children in the following circumstances:
• Any child, aged 6 weeks or above, born to an HIV-positive woman irrespective of whether the woman received antiretroviral therapy in pregnancy
• Any child, 6 weeks or more, who is HIV-positive regardless of symptoms
*Note: All HIV-positive children should be offered cotrimoxazole because they have higher viral loads than adults, progress faster to AIDS and to death compared with adults and at present do not have the same opportunities to access antiretroviral therapy as adults*

Reference [30]

### From policy to practice: scaling up of cotrimoxazole preventive therapy for people living with HIV and impact on early deaths on antiretroviral therapy

Following the adoption of the policy, the HIV department of the MoH wrote a circular with guidelines on CPT drug regimens and individual patient supplies, contraindications, duration of therapy, recruitment, follow-up monitoring and evaluation and drug supply issues. This circular was distributed country-wide for immediate use, and national ART guidelines were eventually updated based on the new evidence [32]. ART treatment cards were modified to incorporate data on use of CPT. Pharmacy dispensing registers for CPT in PLHIV who were not eligible for ART were also developed and printed to track uptake and usage of CPT, and these were placed in pharmacies under the responsibility of pharmacy technicians. A training package was developed, and the CPT policy and guidelines were incorporated into the ARV-HIV related diseases management module that was taught to clinicians and nurses in the country. The policy was also incorporated into other ongoing training courses such as Integrated Management of Childhood Illness (IMCI). Teachers at the various training institutions in Malawi for nurses, clinical officers and medical doctors were made aware of the policy revisions so that they could incorporate them into the curriculum for undergraduate teaching of the management of HIV-related illness. A non-governmental organization assisted the HIV Department in training clinical, nursing and pharmacy staff at all district and mission hospitals in the country, and especially pharmacy technicians on the use and monitoring of CPT.

National forecasting and procurement of CPT needs was integrated into the established practices for ARV drugs. Special packaging of 120 cotrimoxazole tablets per tin was ordered to facilitate 2-month adult dispensing, and thus removing the previous tiresome burden on nurses having to count tablets from 1000-tablet tins. The number of patients receiving CPT is now recorded every quarter as part of the HIV Department's quarterly reports for the country.

As of December 2010, 95% of the 250,987 patients on ART (including HIV-infected TB patients) were on CPT, and a cumulative total of 338,609 patients (pre-ART and ART) had been entered in CPT registers. However, this underestimates the use of CPT as the registers had not been used consistently by all sites [33]. Early mortality on ART has declined considerably. In quarter 2, 2006, 11% of new patients died within the first three months of ART initiation [33]. Early mortality has declined to less than 5% in quarter 4, 2010, according to the routine records [33]. This may be partly due to CPT and also due to the decline in the proportion of patients starting ART in WHO Clinical Stage 4 from 25% in quarter 2, 2005, to about 10% in quarter 4, 2010 [33].

### Lessons learnt

The operational research conducted on HIV testing and CPT, first to HIV-infected TB patients and then to all PLHIV, provides some important lessons about how to successfully integrate operational research into a programme setting. The key stages for this were: initial placement of "operational research" within the programme setting and ensuring senior persons could act as "policy entrepreneurs"; developing relevant research questions; carrying out the research studies; disseminating and publishing the study findings; translating the study findings into action on the ground; and assessing the impact on programme performance. Some of the key lessons learnt, including generic lessons, are illustrated in Table 4, and are further discussed below.

**Table 4:** Generic lessons learnt from operational research with cotrimoxazole preventive therapy in Malawi

Malawi-based experience	General lessons learnt
There were high case fatality rates of TB patients on anti-TB treatment alone, and thus a need for HIV-specific interventions There were high early death rates of people living with HIV starting antiretroviral treatment	Research questions must be relevant to programme needs. Operational research leadership and coordination must be placed within the programme.

Research on cotrimoxazole was endorsed by MoH, and district studies were designed and implemented in conjunction with national programme staff	Research should be endorsed and designed with programme MoH staff in order to increase the probability of findings and recommendations from the study being accepted and implemented

Research was carried out at district or facility level using routine systems; data were collected using registers and treatment cards; all patients were included with no special inclusion and exclusion criteria	Research can and should be effectively carried out within programme settings and routine health services

Key actors or "policy entrepreneurs" in the programmes helped to move forward the process of policy making National meetings were held to engage all stakeholders, to obtain "buy-in" of the results and to get advice and direction as to how to move forward Publication of results in international-peer reviewed journals brought credibility to findings as a result of the peer-review process, and allowed dissemination of results internationally	Key actors or "policy entrepreneurs" must be identified and given the task of moving forward the policy process When research is completed, dissemination must occur nationally, and if judged of wider importance then internationally as well Publication of operational research in peer-reviewed journals adds credibility to the study findings

Clear policy decisions were obtained from MoH about the study findings, and directives given about how to implement the new interventions	Research should influence national policy and practice

Policy documents were prepared and widely distributed through circulars around the country National Guidelines were updated with new evidence and new policy Monitoring tools were prepared and disseminated; drug forecasting was integrated into established processes; training materials were developed and used at different levels; uptake of new interventions were reported in national quarterly reports	Programmes need to implement the new policy and practices Key actors and "policy entrepreneurs within programmes play an important role in this process International guidelines or a road-map need to be developed to better direct the national steps that logically help move research to policy and practice

There was a clear demonstration of impact in reducing case fatality and increasing treatment success in TB patients, and in reducing early death rates in people with HIV starting ART	The ultimate benefit is an impact on programme performance and treatment outcomes

MoH = Ministry of Health; ART = antiretroviral therapy

#### Contextual placement of operational research within a programme setting

Right from the start, the operational research programme was placed within the Malawi NTP with the Programme Director strongly supporting and the National TB Advisor taking responsibility for coordinating the research programme. These two people were the "policy entrepreneurs" (see Table 1), well connected to senior people in the Ministry of Health and to other stakeholders in the health sector such as the Medical School and non-governmental organizations. A similar context prevailed in the HIV/AIDS programme. The small size of the country, the strong support from the Government Ministry of Health for this type of work and the close connections with other key stakeholders in the health sector were important determinants of the success of the operational research. Larger countries with different political and governance systems may find this more difficult.

#### Defining the research questions, getting "buy-in" and using "policy entrepreneurs"

The importance of defining relevant questions for programme and country staff, obtaining "buy-in" from national programme staff and other interested stakeholders at the beginning of a project and having the key actors or "policy entrepreneurs" [34] to push forward the policy-making process cannot be over-emphasised, and these were probably the most important elements of the success of moving this research endeavour through to policy and practice. Without this structure, it is likely that the research would have been published, but without the impact for changing policy or practice. The research questions that were asked were priorities for the programme, and were not set by academic institutions which might have had a different agenda. Furthermore, the results of the various studies were of immense interest to the NTP and to the HIV/AIDS programme, and this ensured that strong linkages were made in getting the research findings to policy at the Ministry of Health and to practice at health facilities in the districts. Important lessons are that operational research should be embedded within a programme structure with a focal point identified, research questions asked from within the programme and a clear budget line set aside to support activities.

#### Disseminating and publishing results

It is important to disseminate and particularly publish results, as the latter lends credibility to the findings [35]. Operational research, if undertaken, is often not written up and submitted for scientific publication, and many of the lessons that could be learnt do not appear in the public domain [36,37]. At country level, it is crucial to have a clear roadmap for dissemination through MoH channels to allow policies to be adopted and the necessary practices that are needed for implementation to be driven forward on the ground.

#### Translating research into policy and practice on the ground

At present, guidelines or a road-map for this process of moving research into policy and practice do not exist at national or international level, and the activities that happen tend to be ad hoc. This should change, and clear, practical steps for dissemination and influencing policy and practice need to be made, based on successful experiences such as those illustrated in this paper.

#### Assessing the impact on programme performance

Ultimately, any change in policy and practice has to benefit patients and the community, and hence the ultimate judge of success is whether treatment outcomes improve or not. It is sometimes difficult to ascribe direct causality in these situations, but that is of secondary concern to programmes where achievement of performance (be it through a direct effect or as an indirect effect of introducing new interventions) has to be the ultimate goal.

### Summary

• In Malawi, high case fatality rates in patients with tuberculosis (TB), who were also co-infected with HIV, and high early death rates in people living with HIV during the initiation of antiretroviral treatment (ART) adversely impacted on treatment outcomes for the national TB and ART programmes respectively.

• District and facility-based operational research was undertaken to assess the effectiveness of cotrimoxazole preventive therapy (CPT) in reducing death rates in TB patients and subsequently patients starting ART under routine programme conditions. Studies showed the beneficial effects of CPT in HIV-infected TB patients and in HIV-infected patients about to start ART, following which the findings were rapidly disseminated nationally at stakeholder meetings convened by the Ministry of Health and internationally through conferences and peer-reviewed scientific publications.

• The Ministry of Health made policy changes based on the available evidence, following which there was countrywide distribution of the updated policy and guidelines. Policy was rapidly moved to practice with the development of monitoring tools, drug procurement and training packages. National programme performance improved, as was demonstrated from routine data, which showed a significant decrease in case fatality rates in TB patients as well as a reduction in early death rates in people with HIV starting ART.

• Key lessons for moving this research endeavour through to policy and practice were the importance of placing operational research within the programme setting, defining relevant questions for programme and country staff, obtaining "buy-in" from national programme staff at the beginning of projects and having key actors or "policy entrepreneurs" to push forward the policy-making process.

## List of abbreviations used

AIDS: acquired immune deficiency syndrome; ART: antiretroviral therapy; CPT: cotrimoxazole preventive therapy; DOTS: directly observed treatment, short course; GFATM: Global Fund to fight AIDS, TB and malaria; HIV: human immunodeficiency virus; IMCI: Integrated Management of Childhood Illness; MoH: Ministry of Health; NTP: national tuberculosis control programme; PLHIV: people living with HIV.

## Competing interests

The authors declare that they have no competing interests.

## Authors' contributions

ADH and RZ had the idea for the paper and wrote the first draft. All authors contributed to further drafts of the manuscript, and all read and approved the final manuscript.

## Ethics Statement

An ethics statement was not required for this work.

## Pre-publication history

The pre-publication history for this paper can be accessed here:

http://www.biomedcentral.com/1471-2458/11/593/prepub
